# Glycosylation status of serum immunoglobulin G in patients with prostate diseases

**DOI:** 10.1002/cam4.662

**Published:** 2016-02-16

**Authors:** Saiko Kazuno, Jun‐ichi, Furukawa, Yasuro Shinohara, Kimie Murayama, Makoto Fujime, Takashi Ueno, Tsutomu Fujimura

**Affiliations:** ^1^ Laboratory of Proteomics and Biomolecular ScienceResearch Support CenterTokyo113‐8421Japan; ^2^ Laboratory of Medical and Functional GlycomicsGraduate School of Advanced Life ScienceFrontier Research Center for Post‐Genome Science and TechnologyHokkaido UniversitySapporo001‐0021Japan; ^3^ Division of UrologyDepartment of MedicineJuntendo University Graduate School of MedicineTokyo113‐8421Japan; ^4^ Present address: Laboratory of Bioanalytical ChemistryTohoku Pharmaceutical UniversitySendai981‐8558Japan

**Keywords:** IgG, mass spectrometry, N‐linked glycan, prostate cancer, PSA

## Abstract

Occurrences of high values in patients with benign prostate disease and low values in patients with highly suspicious cancer have diminished the trustworthiness of prostate‐specific antigen as an early diagnostic marker of prostate cancer. In the search for other complimentary markers, we focused on serum IgG from patients with prostate diseases as well as normal subjects. IgG purified from the sera of normal control subjects and patients with prostate diseases, was digested with peptide N‐glycanase. Released glycans were quantified using MALDI‐time of flight mass spectrometry. We report that *N*‐linked (N‐acetylhexosamine)_2_(deoxyhexose)(mannose)_3_(N‐acetylglucosamine)_2_ was significantly increased in the IgG heavy chains of patients with prostate cancer compared with that of either benign prostatic disease patients or healthy subjects, whereas (hexose)(N‐acetylhexosamine)_2_(deoxyhexose)(mannose)_3_ (N‐acetylglucosamine)_2_ was more abundant in the heavy chains of healthy subjects and benign prostatic disease patients. Thus, an absence of the terminal hexose of N‐linked glycans has been closely connected to the progression of prostate cancer. Furthermore, surface plasmon resonance analyses have revealed that IgG from patients with prostate cancer has a decreased binding for *Sambucus nigra* lectin, compared with that from the benign prostatic disease patients or from normal subjects, suggesting lower levels of (N‐acetylneuraminic acid)(*α*2‐6)galactose/N‐acetylgalactosamine groups in the N‐linked glycans of patient IgG. Meanwhile, wheat germ agglutinin binding to IgG of the cancer group was significantly larger than that for the benign prostatic disease group but smaller than that for normal subjects. Our study indicates that the glycosylation changes in IgG can become useful diagnostic parameters for prostate cancer.

## Introduction

Cancer‐associated chronic inflammation upregulates the production of inflammatory cytokines, such as (IL, interleukin) IL‐6, TNF‐*α* (tumor necrosis factor,TNF), and IL‐1*β* 1. The released cytokines stimulate pleiotropic signaling pathways to modify the gene expressions of proliferating cancers. One important consequence of the effects is an increased expression of acute phase proteins (APPs) including *α*1‐antitrypsin, *α*1‐acid glycoprotein, C‐reactive protein, haptoglobin, serine protease inhibitors, etc 2, 3, 4, 5.

Acute phase proteins are a group of plasma proteins, many of which are glycosylated. Another consequence is the altered and/or hyperactive glycosylation of tissue plasma membrane proteins and proteins that are secreted from malignant tissues 6, 7, 8. These two effects frequently occur simultaneously and many studies have reported cancer‐associated changes in the glycosylation of APPs. It should also be noted that depending on the regions or tissues where cancer develops, diverse types of glycosylation of serum proteins could occur. In fact, many clinics have established carbohydrate antigens such as CA125, CA19‐9, CEA, and Her2/neu as cancer biomarkers 7, 8, 9, 10, 11.

Prostate cancer is ranked as the second most‐frequent cause of cancer death among men aged 60–80 years 12. Current early diagnosis of prostate cancer depends on a measurement of the serum level of prostate‐specific antigen (PSA). PSA is a glycoprotein that is released from the prostate and is classified as a member of the kallikrein‐related peptidase family. The serum level of PSA in normal healthy men is ≤4.0 ng/mL 13. However, relatively lower values for PSA levels (4–10 ng/mL) have also been detected in some patients who bear aggravated prostate cancer. Conversely, high values for serum PSA levels (≥10 ng/mL) are frequently recorded in some patients with benign prostatic disease (BPD) 14. These observations indicate that the use of multiple markers for diagnosing early stages of prostate cancer is very important. We and other groups have disclosed that the glycosylation status of serum proteins in patients with prostate cancer is closely correlated with cancer progression. Saito et al. found that intense immunoreactivity of the monoclonal antibody RM2, which recognizes *β*1,4‐GalNAc‐disialyl‐Lc_4_, is strongly associated with a large tumor volume and advanced stages of prostate cancer 15. Subsequent analyses by this group revealed that an increase in the RM2 reactivity to haptoglobin, a representative of the acute phase proteins (APP), is a key determinant for more specific and sensitive diagnoses of early‐stage prostate cancer 16. In the meantime, mass spectrometric analyses by Fujimura et al. have revealed that triantennary glycans attached to the N207 and N211 of haptoglobins were more fucosylated in patients with prostate cancer compared with those found in BPD and normal controls 17. Independently, Nakano et al. have also identified di‐fucosylated tetra‐antennary N‐glycans of haptoglobin in patients with pancreatic cancer 18, 19. Haptoglobin fucosylation has also been detected in hepatocellular carcinoma 20. Thus, fucosylation and universal glycosylation alteration of serum haptoglobins seems to be representative of diverse cancers. Fucosylation of haptoglobins has also been confirmed by lectin blotting using *Aleuria aurantia* lectin (AAL) in patients with prostate cancer 19, 21. More recently, we conducted surface plasmon resonance (SPR) analysis based on multisequential analysis using *Sambucus nigra* lectin (SNA‐1), AAL, and *Phaseolus vulgaris* lectin (PHA‐L_4_) to assess the glycosylation of haptoglobin, and found that SNA‐1 could effectively detect Neu5Ac*α*2,6 in the biantennary glycans of haptoglobin in patients with prostate cancer 22. During the course of this study, we noticed that some lectins reacted with a 50 kDa band of sera from the patients with prostate diseases, as well as those from normal healthy subjects, which we suspected might be attributable to IgG heavy chains. In contrast to haptoglobin, alteration of IgG‐associated N‐glycans has not been fully investigated in relation to prostate cancer or BPD 5. In this study, we focused on the glycosylation status of IgG in patients with prostate cancer compared with that in BPD patients and in normal healthy subjects using mass spectrometry and SPR analysis.

## Materials and Methods

### Reagents

*Aleuria aurantia* lectin‐biotin and wheat germ hemagglutinin (WGA) were purchased from J‐OIL MILLS (Tokyo, Japan). *Sambucus nigra* lectin (SNA‐1) and its horseradish peroxidase (HRP) and biotin derivatives were obtained from EY Laboratories, Inc. (San Mateo, CA). Mouse monoclonal antibodies against sialyl‐Lewis^a^ (CA19‐9, NS19‐9) were purchased from Wako Pure Chemical Industries (Osaka, Japan). Anti‐rabbit IgG‐HRP conjugate and anti‐mouse IgG‐HRP conjugate were purchased from Jackson ImmunoResearch Laboratories, Inc. (West Grove, PA). BlotGlyco beads were obtained from Sumitomo Bakelite Co., Ltd. (Tokyo, Japan).

### Serum samples

Serum samples of 25 patients with prostate cancer (malignant), 28 patients with BPD, and 10 control healthy volunteers, were obtained from the Division of Urology, Department of Medicine, Juntendo University School of Medicine, as described previously 22. The experimental protocol was approved by the Ethics Committee of Juntendo University Hospital and Juntendo University School of Medicine (No. 20‐37‐2) and conforms to the provisions of the Declaration of Helsinki in 1995. A signed consent form was obtained from each subject. The pathological hallmarks of these patients are described in Table 1.

**Table 1:** The Gleason scores and prostate‐specific antigen (PSA) values in the serum of patients with prostate cancer (Prostate Cancer), patients with benign prostate diseases (BPD), and normal healthy control subjects (Healthy Control)

Sample number	Gleason Score	PSA ng/mL	Protein conc. mg/mL
Prostate Cancer			
U106	4 + 5	300.5	61.5
U80	4 + 4	63.64	70.8
U81	4 + 4	5.86	63.6
U102	4 + 4	7.52	69.6
U103	4 + 4	4.92	65.4
U124	4 + 4	25.92	64.4
U77	4 + 3	8.01	69.9
U111	4 + 3	6.08	69.7
U120	4 + 3	18.73	83.7
U12	3 + 4	9.01	73.5
U47	3 + 4	25.07	67.5
U78	3 + 4	5.01	67.0
U86	3 + 4	10.71	65.7
U87	3 + 4	12.27	65.7
U108	3 + 4	56.82	68.6
U112	3 + 4	8.70	67.0
U113	3 + 4	20.51	75.7
U115	3 + 4	5.92	65.6
U118	3 + 4	20.90	69.7
U130	3 + 4	8.15	74.2
U79	3 + 3	7.54	53.7
U104	3 + 3	8.87	66.5
U109	3 + 3	12.25	59.7
U141	3 + 3	9.46	70.2
U143	3 + 2	17.24	81.3
BPD			
U44		6.71	64.4
U57		4.95	68.3
U68		10.84	65.6
U72		3.66	61.7
U74		7.41	64.6
U75		4.68	61.6
U76		5.72	58.5
U83		4.75	64.0
U84		6.72	65.0
U101		16.89	66.7
U105		7.95	63.6
U114		18.60	72.2
U119		6.02	74.1
U125		17.43	63.2
U126		11.78	68.9
U127		7.77	76.4
U128		6.65	65.6
U129		6.43	79.3
U136		4.13	90.0
U137		6.47	86.4
U138		6.10	63.6
U140		5.98	67.1
U142		7.24	93.6
U144		6.60	87.8
U145		6.05	69.3
U147		6.05	50.7
U148		4.62	63.4
U149		13.52	63.9
Healthy control			
UC1		0.98	67.8
UC2		0.81	67.4
UC3		2.04	71.1
UC4		0.64	65.1
UC5		0.45	77.0
UC6		1.01	69.7
UC7		2.13	68.5
UC8		2.31	67.0
UC9		0.88	66.2
UC10		0.73	68.3

### Glycosylation analysis of serum immunoglobulin by MALDI‐TOF mass spectrometry

Serum IgG was purified using Protein G‐Sepharose (Fast Flow, GE Healthcare Amersham Place, UK) according to the manufacturer's protocol. Purified IgG or IgG heavy chains separated by SDS‐PAGE 23 were reductively alkylated and digested with trypsin 17. Then, *N*‐glycans were enzymatically liberated from trypsin‐digested peptides using PNGase‐F( peptide N‐glycanase). The released N‐glycans were purified using BlotGlyco beads equipped in a BIOFELLOW^®^ glyco instrument (SYSTEM INSTRUMENTS Co., Ltd., Tokyo, Japan) 17. Sialic acid residues of *N*‐glycans were methylesterified and labeled with aoWR [N^a^‐((aminooxy)acetyl) tryptophanylarginine methylester] 17. Then, *N*‐glycans were mixed with 2,5‐dihydroxybenzoic acid (10 mg/mL in water) and subjected to MADLI‐TOF (time of flight) mass measurements using an Ultraflex TOF mass spectrometer equipped with a pulsed ion extraction system (Bruker Daltonics GmbH, Bremen, Germany). Quantification of *N*‐glycans was performed, as described previously 17.

### Quantification of serum PSA and the assessment of a pathological state

Serum PSA was determined using an AxSYM analyzer (Abbott Laboratories, Abbott Park, IL). Prostate cancer and BPD were diagnosed based on histopathological examination of prostate tissues, and prostate malignancies were graded using a Gleason score as described previously 22.

### SPR analysis

Surface plasmon resonance (SPR) analysis was performed using a BIAcore2000 (GE Healthcare). Anti‐human IgG Fc antibody was immobilized on a sensor chip C1 according to a previously reported method with slight modification 22. Briefly, the instrument with the docked sensor chip that had been conditioned with HBS‐EP (10 mmol/L HEPES pH 7.4, 150 mmol/L NaCl, 3 mmol/L EDTA and 0.005% surfactant P20) was washed with a washing buffer (50 mmol/L glycine/NaOH, pH 9.5, and 0.3% Triton X‐100). Subsequently, the surface of the sensor was activated with a mixture of NHS (*N*‐hydroxysuccinimide) and EDC (*N*‐ethyl‐*N'*‐(3‐dimethylaminopropyl) carbodiimide) for 7 min, and coupled with 25 *μ*g/mL of anti‐human IgG Fc in 10 mmol/L acetate buffer, pH 4.5. The remaining residues on the sensor were deactivated with 1 mol/L ethanolamine hydrochloride, pH 8.5, for 15 min. This procedure immobilized about 1000 resonance units (RU). Of 4 flow cells, the first (Fc1) immobilized no ligand and was used as a blank cell.

Serum samples (1 mg protein/mL) in running HBS‐EP buffer containing 0.2 mg/mL bovine serum albumin (BSA), were filtered through 0.45 *μ*m ultrafree‐MC centrifugal filter devices (Millipore, Billerica, MA). The filtrate was diluted again to 100 *μ*g/mL using the running buffer with or without lectin (20 *μ*g/mL). Binding analysis was performed for 4 min at a flow rate of 10 *μ*L/min and each measurement was performed in duplicate. The results were evaluated using BIA evaluation software 4.1. The differences in the binding of RU between IgG premixed with and without lectin were compared among patients with prostate cancer, BPD, and healthy subjects.

### Lectin blotting

Lectin blotting was performed according to a previously published procedure 22. Namely, purified IgG was separated in 10% SDS‐PAGE gels and subsequently transferred onto polyvinylidene difluoride (PVDF) membrane. The membrane was blocked with 3% BSA and then washed three times with 20 mmol/L Tris‐HCl (pH 7.5)‐0.15 mol/L NaCl‐0.05% Tween20 (TTBS). The membrane was incubated overnight at 4°C with 2 *μ*g/mL AAL‐biotin. After washing three times with TTBS, the membrane was incubated with anti‐biotin‐horseradish peroxidase conjugate for 1 h. The membrane was extensively washed with TTBS and the reactivity was developed by ECL‐Plus (GE Healthcare) according to the manufacturer's protocol.

### Cytokine/chemokine and cancer biomarker measurement

Cytokine/chemokine and conventional cancer biomarkers in serum samples were measured using a Luminex 200 multiplex assay instrument with xPONENT 3.1 software (Luminex Corporation, Austin, TX) that featured a milliplex map kit of human cytokine/chemokine 14plex premix beads kit and a cancer biomarker panel kit (Merk Millipore, MA). Each serum sample was assayed in duplicate according to the procedure provided by the manufacturers. The data quantification was processed using OriginPro 9.1 software (OriginLab Corporation, Northampton, MA).

### Statistical analysis

All data are presented as the means ±SD. The statistical differences between the two groups were evaluated using a two‐tailed Student's *t* test. A *P* value of less than 0.05 was considered statistically significant. For the principal component analysis, the data were calculated using OriginPro 9.1 software and Easy PCA (http://hoxom-hist.appspot.com/pca.html).

## Results

### Pathological profiles of patients with elevated serum inflammatory cytokines

Table 1 summarizes Gleason scores and PSA levels of the sera from patients with prostate cancer (*n* = 25), patients with BPD (*n* = 28), and normal healthy subjects (*n* = 10) used in this study. The Gleason score in 20 of 25 patients with prostate cancer was more than 7 (e.g., 4 + 3, 3 + 4, 4 + 4, or 4 + 5) and five or six in the remaining five patients. In some patients with prostate cancer, serum PSA levels were relatively high and very high (300 ng/mL) in one patient (U106) with Gleason score of 4 + 5, while remaining 13 of 25 patients with prostate cancer had low serum PSA values (<10 ng/mL). On the other hand, six of the 28 patients with BPD had comparatively high PSA values (>10 ng/mL). Serum PSA levels were within the normal range (<3 ng/mL) in all control subjects.

**Figure 1:**
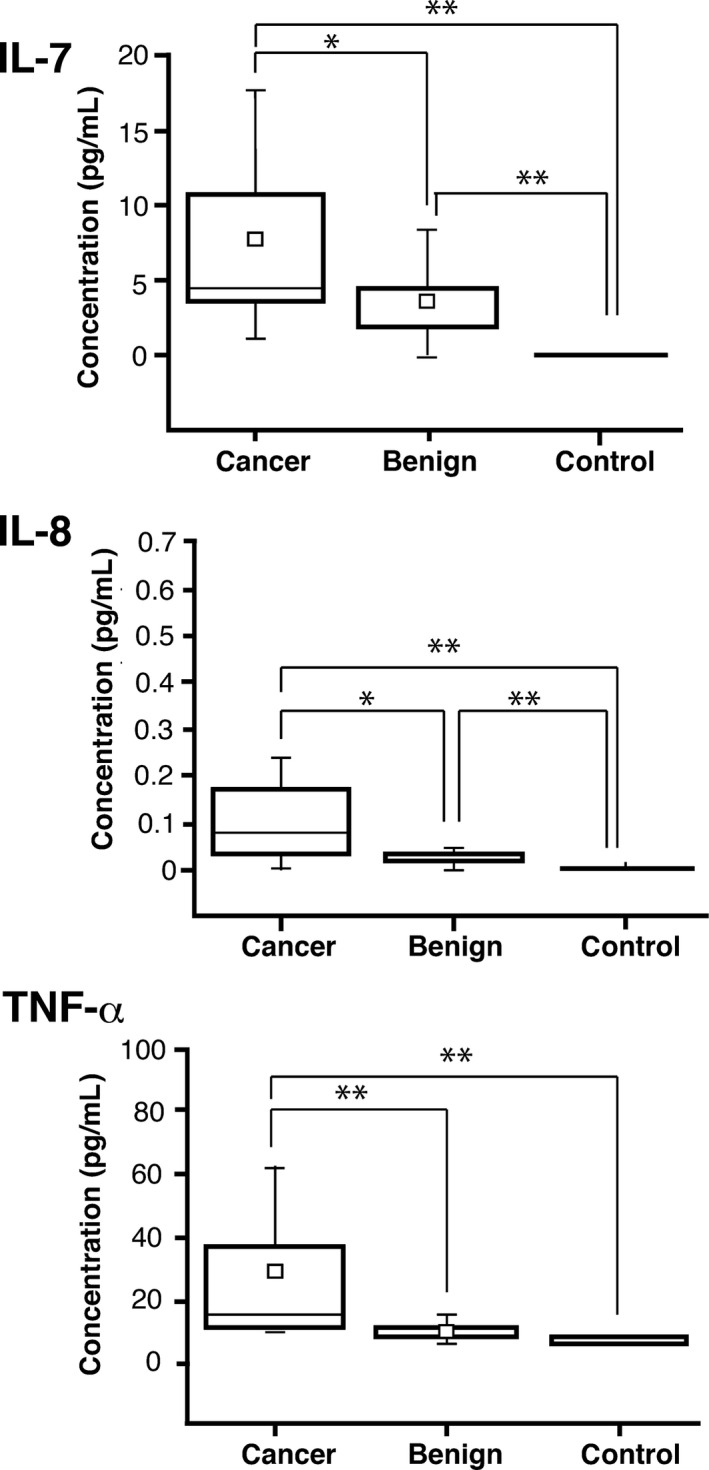
Box graphs of (interleukin,IL) IL‐7, IL‐8, and tumor necrosis factor (TNF)‐*α* concentrations in serum samples determined by Luminex 200 multiplex beads assay. The small open square represents an average. The horizontal line in the box represents the median value (Cancer: *n* = 17, Benign: *n* = 17, Normal: *n* = 5). *P < 0.05, **P < 0.01.

It has been reported that serum levels of representative cytokines and chemokines are known to be upregulated in prostate diseases 24, 25, 26, 27. We randomly chose the sera from 17 patients with prostate cancer, 17 patients with BPD, and five healthy subjects and determined serum cytokines using Luminex 200 multiplex assay. As Figure 1 shows, serum IL‐7, IL8, and TNF‐*α* levels were all significantly higher in patients with prostate cancer than those found in either BPD patients or normal healthy subjects with *P* values of less than 0.05 (Fig. 1). Similar tendencies were recognized for the serum levels of IL‐6, IL‐10, and MIF (macrophage migration inhibitory factor), although the differences were not statistically significant (data not shown). No other cytokines or cancer markers, such as MCP‐1 (monocyte chemotactic protein‐1), GM‐CSF (granulocyte macrophage colony‐stimulating factor), IGF‐II, exhibited differences between the prostate disease groups and the normal healthy group (data not shown). These data are basically consistent with the data published previously 24, 25, 26, 27.

### Glycosylation analysis of the serum IgG of prostate cancer and BPD

Cancer‐induced changes in the glycosylation status of haptoglobin, *α*1‐acid glycoprotein, and *α*1‐anti‐chymotrypsin, have been fully investigated, however, relatively little has been investigated pertaining to the glycosylation of serum major resident proteins such as immunoglobulins. In the previous studies on serum haptoglobin in prostate cancer 17, 22, we frequently noticed that some lectins, including AAL and WGA, strongly recognized a ~50 kDa band of sera from patients with prostate cancer as well as BPD in lectin blotting. Figure S1A lists representative data of an example showing the reactivity of AAL to purified IgG heavy chains from normal healthy humans, patients with prostate cancer, and patients with BPD, as described in the Materials and Methods section. The antibody strongly reacted with 50 kDa heavy chains, whereas only a dim signal could be seen with 25 kDa light chains. Quantitative densitometry revealed more intense reactivity of AAL to IgG from patients with prostate cancer than that from BPD or normal subjects (Fig. S1B).

We next focused more precisely on the glycosylation status of serum IgG heavy chains. As the carbohydrate antibodies used in western blotting are specific for glycan groups, but not for particular chain structures of glycans, it was intriguing to examine whether or not IgG heavy chain‐associated glycans have structural differences among patients with prostate diseases. Heavy chains of purified serum IgG were subjected to reductive alkylation and digested with trypsin. The digests were treated with PNGase‐F to liberate *N*‐glycans and the released N‐glycans were then subjected to MALDI‐TOF mass measurements using an Ultraflex TOF mass spectrometer.

**Figure 2:**
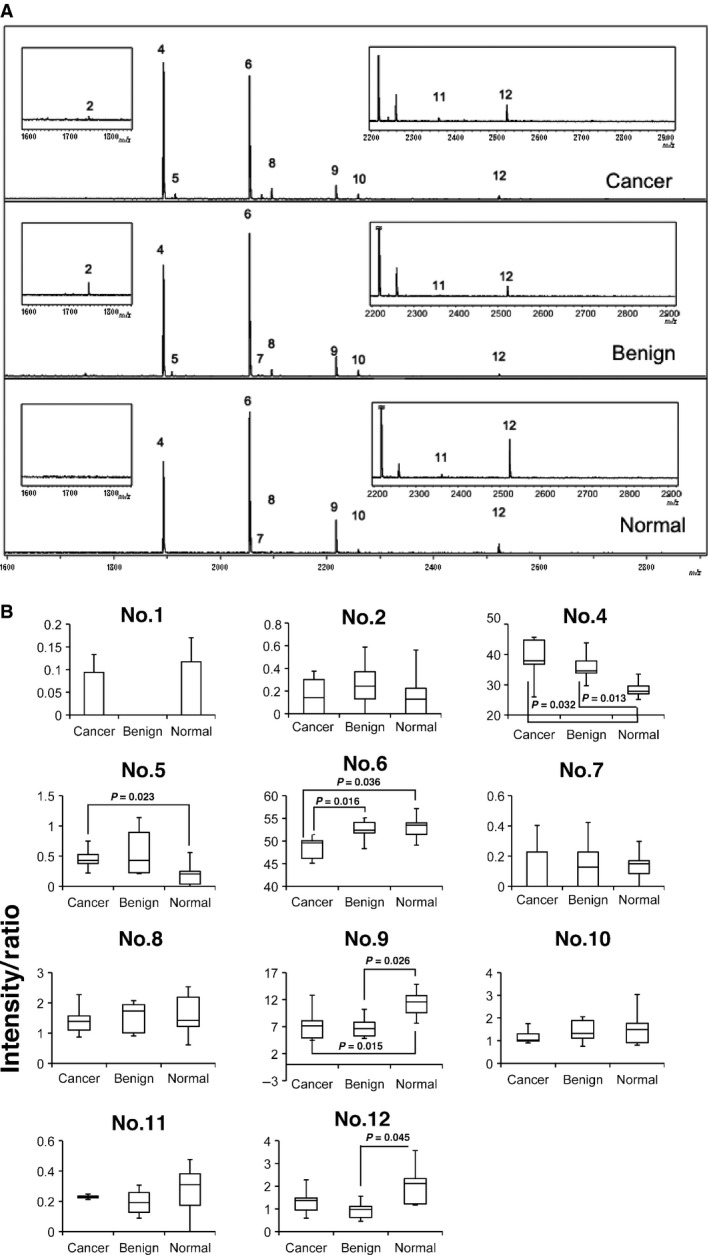
Quantification of N‐linked glycans associated with serum IgG heavy chains from patients with prostate cancer, patients with benign prostatic diseases, and normal healthy subjects. (A). MALDI‐TOF mass spectra showing N‐glycan profiles of serum IgG from patient with prostate cancer (Cancer), patient with benign prostatic disease (Benign), and normal healthy subject (Normal). The data shown are representative of five separate experiments in each experimental group. (B). Quantitative analysis of MALDI‐TOF mass spectra. Quantification was performed with separated 12 peaks (*n *= 5). Note that two glycans, No. 4 and No. 6, which were consistently found associated with IgG heavy chains, correspond to [(HexNAc)_2_(Deoxyhexose)_1_ + (Man)_3_(GlcNAc)_2_] and [(Hex)_1_(HexNAc)_2_(Deoxyhexose)_1_ + (Man)_3_(GlcNAc)_2_], respectively. Although peaks 1, 3, and 13 were detected mechanically, their content (intensity) was too small to quantify. The intensity/ratio as described in reference 17 was calculated for each experimental group.

The list presented in Table 2 and Figure 2A summarizes the glycosylation structures of *N*‐linked glycans that were derived from the heavy chains of purified serum IgG. Quantification of these glycan structures revealed that significantly more of (HexNAc) N‐acetylhexosamine [(HexNAc)_2_ (Deoxyhexose)_1_ + (Man)_3_ (GlcNAc)_2_] (peak 4) was found in a IgG heavy chain from the sera of a patient with prostate cancer than could be detected either in the heavy chain of patients with BPD or in that of normal control subjects (Fig. 2). On the other hand, more [(Hex)_1_(HexNAc)_2_(Deoxyhexose)_1_ + (Man)_3_(GlcNAc)_2_](peak 6) was found associated with a heavy chain from the serum immunoglobulin from normal subjects and BPD patients than that from patients with prostate cancer (Fig. 2B, Table 2). Hence, the presence or absence of the terminal hexose of *N*‐glycans could be a key determinant in the diagnosis for prostate cancer. As for other glycans, there were no quantitative differences among the IgG heavy chains from either the patients or the normal healthy subjects.

**Table 2:** List of N‐linked glycan structures from IgG heavy chains, as determined by MALDI‐TOF (time of flight) mass spectrometric analysis (Fig. 2)

No.	Mol. Mass	Assigned composition	Relative amount (mean ± SD)		
			Cancer	Benign	Normal
1	1689.8237	(HexNAc)_1_(Deoxyhexose)_1_ + (Man)_3_(GlcNAc)_2_	0.05 ± 0.06	0.00 ± 0.00	0.06 ± 0.08
2	1746.7541	(HexNAc)_2_ + (Man)_3_(GlcNAc)_2_	0.16 ± 0.17	0.27 ± 0.23	0.09 ± 0.11
3	1851.8935	(Hex)_1_(HexNAc)_1_(Deoxyhexose)_1_ + (Man)_3_(GlcNAc)_2_	0.00 ± 0.00	0.00 ± 0.00	0.00 ± 0.00
4	1892.8917	(HexNAc)_2_(Deoxyhexose)_1_ + (Man)_3_(GlcNAc)_2_	38.23 ± 7.89	34.01 ± 3.36	27.37 ± 1.84
5	1908.8931	(Hex)_1_(HexNAc)_2_ + (Man)_3_(GlcNAc)_2_	0.39 ± 0.13	0.58 ± 0.42	0.12 ± 0.12
6	2054.9466	(Hex)_1_(HexNAc)_2_(Deoxyhexose)_1_ + (Man)_3_(GlcNAc)_2_	48.47 ± 2.71	53.33 ± 1.54	53.03 ± 3.00
7	2070.9392	(Hex)_2_(HexNAc)_2_ + (Man)_3_(GlcNAc)_2_	0.13 ± 0.18	0.16 ± 0.18	0.10 ± 0.08
8	2095.9783	(HexNAc)_3_(Deoxyhexose)_1_ + (Man)_3_(GlcNAc)_2_	1.48 ± 0.67	1.53 ± 0.54	1.60 ± 0.77
9	2216.9994	(Hex)_2_(HexNAc)_2_(Deoxyhexose)_1_ + (Man)_3_(GlcNAc)_2_	6.16 ± 1.74	6.95 ± 2.17	11.27 ± 2.79
10	2258.0164	(Hex)_1_(HexNAc)_3_(Deoxyhexose)_1_ + (Man)_3_(GlcNAc)_2_	1.05 ± 0.17	1.42 ± 0.54	1.24 ± 0.46
11	2360.0530	(Hex)_1_(HexNAc)_2_(Deoxyhexose)_1_(NeuAc)_1_ + (Man)_3_(GlcNAc)_2_	0.23 ± 0.00	0.19 ± 0.09	0.27 ± 0.19
12	2522.0977	(Hex)_2_(HexNAc)_2_(Deoxyhexose)_1_(NeuAc)_1_ + (Man)_3_(GlcNAc)_2_	1.09 ± 0.41	0.94 ± 0.43	2.08 ± 0.98
13	2828.2742	(Hex)_2_(HexNAc)_2_(Deoxyhexose)_1_(NeuAc)_2_ + (Man)_3_(GlcNAc)_2_	0.00 ± 0.00	0.00 ± 0.00	0.00 ± 0.00

GlcNAc: N‐acetylglucosamine; Man: mannose; Neu5Ac: N‐acetylneuraminic acid; GalNAc: N‐acetylgalactosamine; HexNAc, N‐acetylhexosamine.

### Reactivity of patient serum IgG to SNA‐1 and WGA lectins determined by SPR analysis

**Figure 3:**
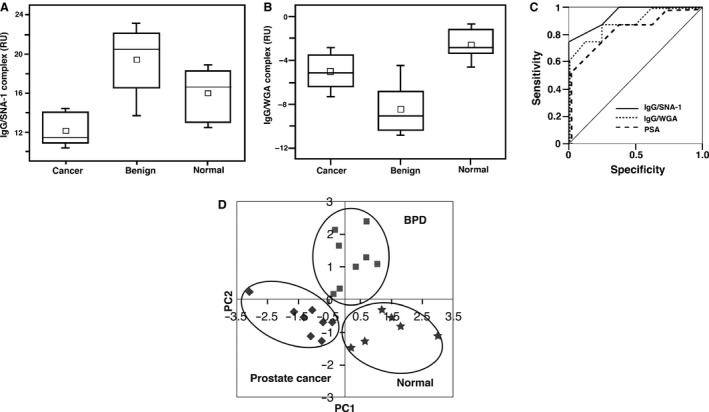
The distribution of the amount of IgG bound with SNA‐1 (A) or wheat germ hemagglutinin (WGA) (B) in the sera of patients, as measured by surface plasmon resonance (SPR) analysis. The small open square represents an average. The horizontal line in the box represents the median value. In the case of IgG bound with SNA‐1 (A), there was a significant difference between the prostate cancer group and the control or BPD (benign prostatic disease) groups with *P* values of 0.0002 and 0.016, respectively (*n* = 12 for prostate cancer group, *n* = 13 for BPD group, *n* = 7 for normal control subjects). In the case of IgG bound with WGA (B), the mean RU of the cancer group was significantly larger than that of BPD (benign prostatic disease) with a *P* value of 0.03, but smaller than that of normal subjects with a *P* value of 0.003 (*n* = 9 for prostate cancer group, *n* = 9 for BPD group, *n* = 7 for normal control group). From the ROC curve (C), the area under the curve (AUC) value was 0.95 for IgG/SNA‐1, represented as a solid line, and was 0.88 for IgG/WGA, represented as a dotted line. Meanwhile, the AUC value for prostate‐specific antigen (PSA), a conventional diagnostic marker, represented as a broken line, was 0.84. The results of the principal components analysis (PCA) were plotted (D). The symbols represent the following: the diamond; prostate cancer patients (*n* = 8), the square; benign prostatic disease (BPD) patients (*n* = 8), the star; the normal subjects (*n* = 6). The four elements consisting of IgG‐SNA‐1, IgG‐WGA, haptglobin‐SNA‐1 and PSA clearly segregate the cancer patients, the BPD patients, and the normal subjects.

We previously conducted multi‐sequential SPR analysis to estimate the glycosylation status of haptoglobin in the sera of patients with prostate diseases using SNA‐1, AAL, and PHA‐L_4_ lectin 22. Adapting this technique, we focused on the SPR analysis, which involved the use of anti‐IgG as a ligand and diluted serum premixed with lectin for efficient detection as an analyte. Figure 3 shows the distribution patterns of the binding amounts of either SNA‐1 or WGA (Fig 3A and B). The binding RU of lectin to IgG were calculated by subtracting the binding amount of IgG without lectin from that of the amount of the IgG‐lectin complex that was generated by the premixing with lectin. As clearly shown, the mean RU difference by SNA‐1 of the prostate cancer group was significantly smaller than that of either the control or the BPD group with *P* values of 0.0002 and 0.016, respectively (Fig. 3A). Meanwhile, the mean RU difference of the WGA binding to an IgG of the cancer group was significantly larger than that for the BPD group with a *P* value of 0.03, but it was smaller than that for normal subjects with a *P* value of 0.003 (Fig. 3B). Thus, the data were distinct from that obtained with haptoglobin, wherein SNA‐1 bound to the haptoglobin in the prostate cancer group with an affinity that was higher than its affinity for binding to the haptoglobin of either the BPD group or the normal control group 22. The ROC curve showed how the IgG‐lectin detection method using SPR analysis can be as valuable as, or even slightly superior to, a diagnosis using the PSA value – the areas under the curve (AUC) were 0.95, 0.88 and 0.84 for the IgG‐SNA‐1, IgG‐WGA and PSA, respectively (Fig. 3C). The RU data were further subjected to principal component analysis, as described in the Materials and Methods section (Fig. 3D). The amount of SNA‐1 bound to haptoglobin in the serum (haptoglobin‐SNA‐1) was measured according to a previously reported method 22. The principal component scores of IgG‐SNA‐1, IgG‐WGA, haptoglobin‐SNA‐1, and PSA in the two‐dimensional X‐Y plot clearly segregated the cancer patients, the BPD patients and the normal subjects, which proves the validity of SNA‐1 and WGA as independent clinical markers for prostate diseases.

## Discussion

It is well known that even the dominant diagnostic marker, the PSA, is not reliable for diagnosing prostate cancer. As for the serum specimens used in this study, those from one half of patients with prostate cancer had low serum PSA values (<10 ng/mL), whereas those from ~20% of the patients with BPD had relatively high PSA values (>10 ng/mL). Definitive diagnosis could only be achieved by histopathological examination (Gleason score) of the prostate tissue. Meantime, IL‐7, IL‐8, and TNF‐*α* were significantly upregulated in patients with prostate cancer compared with patients with BPD and normal control subjects. Elevated expression of IL‐7 and IL‐15 in prostate tissues and its increased serum level was confirmed in patients with early‐stage prostate cancer 25. IL‐8 induces the expression of chemokine receptor CXCR7, which stimulates EGF signaling to promote prostate cancer growth 26. TNF‐*α* enhances the motility and invasiveness of prostate cancer cells (LNCaP) through increased binding to selectins 27. Thus, our findings of the increased levels of IL‐7, IL‐8, and TNF‐*α* (Fig. 1) in patients with prostate cancer are consistent with characteristic features of prostate cancer reported previously.

Use of multiple markers that are complementary to the PSA is recommended for diagnosing the early stages of prostate cancer. The data of lectin blotting that AAL reacted more strongly with the serum IgG heavy chains of patients with prostate diseases than those of BPD as well as healthy control subjects (Fig. S1) attracted our attention. It has been found that altered N‐glycosylation occurs in serum IgG as well as *α*1‐acid glycoproteins in ovarian cancer 28. We reasoned that it is important to characterize glycosylation structures of IgG in prostate diseases, which have been relatively little understood in contrast to those previously clarified with other APPs, including *α*1‐acid glycoprotein, haptoglobin, and antichymotrypsin sampled from patients with various cancers including prostate cancer 6, 15, 16, 17, 18, 19, 20, 28, 29. We explored the detailed glycan structures of serum IgG using MALDI‐TOF mass spectrometry and SPR analysis to measure the specific binding of lectin to N‐glycans, anticipating some clues to find more reliable biomarker(s) of prostate cancer.

Serum IgG molecules possess a conserved N‐glycosylation site in the Fc region and a variable glycosylation site in the Fab region 7, 30, 31. The conserved N‐glycosylation initiates from Asn297 of the Fc region, and expands to construct various biantennary complex structures with either the presence or absence of sialic acid, bisecting N‐acetylglucosamine, etc. 30, 31 Glycosylation of the Fc domain is essential for the interaction between the Fc domain and IgG receptors such as Fc*γ*, Fc*γ*RII, and Fc*γ*RIII 28. Conserved N‐glycans in the Fc regions are known to be frequently altered in various diseases, such as osteoarthritis and rheumatoid arthritis 30, 31. Increased fucosylation and decreased galactosylation has also been found in the serum IgG of patients with ovarian cancer 32.

MALDI‐TOF mass spectrometry measurements have clearly revealed that [(HexNAc)_2_(Deoxyhexose)_1 _+ (Man)_3_(GlcNAc)_2_] (GlcNAc, N‐acetylglucosamine) is significantly increased, whereas [(Hex)_1_(HexNAc)_2_(Deoxyhexose)_1_ + (Man)_3_(GlcNAc)_2_] is decreased, in the serum IgG of patients with prostate cancer compared with BPD patients and normal healthy subjects (Fig. 2). The quantified glycans can be assigned to the biantennary N‐glycan structure of the Fc region; deoxyhexose and HexNac, which presumably correspond to fucose and GlcNAc, respectively, attach to the biantennary core domain comprising (Man)_3_(GlcNAc)_2_. Thus, the presence or absence of one hexose (Hex) that most likely corresponds to the galactose attached to GlcNac on either branch of the biantennary glycan may be a key determinant in distinguishing prostate cancer from either a case of BPD or a normal control.

In another approach using SPR analysis, both SNA‐1 and WGA binding to N‐glycans of serum IgG resulted in sufficient RU increases with statistically significant differences between the normal group and the pathological groups with prostate diseases. The binding RU ratio of SNA‐1 in patients with prostate cancer was significantly smaller than that in patients with BPD or in normal subjects. SNA‐1 is most sensitive to both the (Neu5Ac)(*α*2‐6)Gal and the (Neu5Ac)(*α*2‐6)GalNAc groups of N‐glycans. The quantification of three Neu5Ac‐containing glycans (Table 2, No. 11~No. 13) using MALDI‐TOF mass spectrometry showed no decreases in patients with prostate cancer compared with decreases in BPD patients and in normal subjects, which suggests that the linkage mode of sialic acid(s) (i.e. *α*2–3 or *α*2–6) varies in patients with prostate cancer. Indeed, recent reports have shown that *α*2–3 sialylation in serum N‐glycome is significantly increased in prostate cancer. This compares to benign prostate hyperplasia, though proteins attached with *α*2–3 sialylated N‐glycans were not identified 33, which demonstrates the usefulness of our glycomic approach based on the complementary techniques of structurally intensive mass spectrometric analysis and molecular recognition. WGA binding to serum IgG in patient groups with prostate cancer elicited increases in the binding RU ratio of WGA to IgG, with a magnitude that was larger than that of patients with BPD, but smaller than that of the normal group. It is noteworthy that the principal component analysis supported both the binding RU ratio of lSNA‐1 to IgG and that of WGA to IgG, both of which were well segregated in the two‐dimensional distribution plots and in the ROC curves. Also, the two lectins were useful as independent clinical markers along with PSA to distinguish the normal healthy group from the prostate disease groups. In conclusion, our data strongly supported the use of multiple clinical parameters that use distinct approaches to target different molecules of patient sera in the diagnosis of prostate diseases, rather than relying on a single clinical marker.

## Conflict of Interest

The authors have no conflict of interest.

## Supporting information


**Figure S1.** A. AAL lectin blotting analysis of IgG purified from sera of normal healthy control subjects (Normal), patients with benign prostatic disease (BPD), or the patients with prostate cancer (Cancer).
