# Supplementary figures and images for: Glycosylation status of serum immunoglobulin G in patients with prostate diseases
Source: Cancer Med. 2016 Feb 16;5(6):1137–46. doi: 10.1002/cam4.662 (PMC4924372; doi:10.1002/cam4.662)

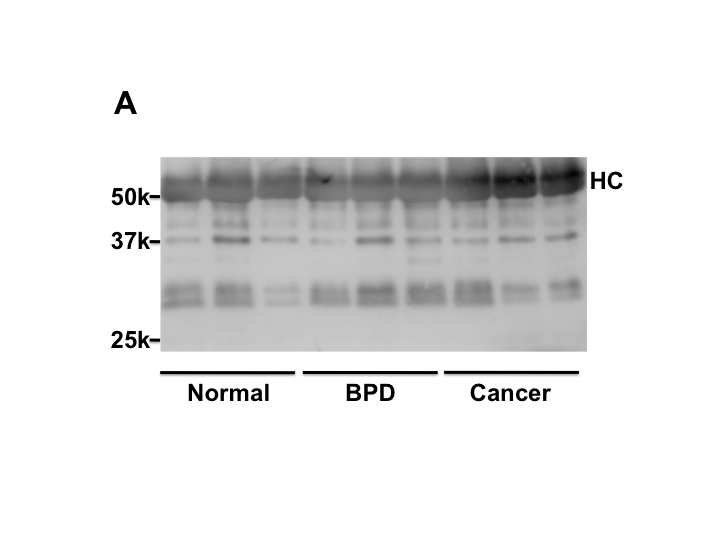

Supplement: Supplementary file 1 — Figure S1. A. AAL lectin blotting analysis of IgG purified from sera of normal healthy control subjects (Normal), patients with benign prostatic disease (BPD), or the patients with prostate cancer (Cancer). [file CAM4-5-1137-s001.tif]
